# Re-esterified DHA improves ventilatory threshold 2 in competitive amateur cyclists

**DOI:** 10.1186/s12970-020-00379-0

**Published:** 2020-10-21

**Authors:** Vicente Ávila-Gandía, Antonio Torregrosa-García, Antonio J. Luque-Rubia, María Salud Abellán-Ruiz, Desirée Victoria-Montesinos, F. Javier López-Román

**Affiliations:** 1 Sports Physiology department, San Antonio Catholic University of Murcia (UCAM), Avenida de los Jerónimos, 135, Guadalupe (Murcia), 30107 Spain; 2 Biomedical Research Institute of Murcia (IMIB-Arrixaca), Murcia, 30120 Spain

**Keywords:** Docosahexaenoic acid, Cycling, Ergogenic effect, Performance, Omega-3

## Abstract

**Background:**

Fish oils were studied as ergogenic aids in a number of mixed physical trial designs showing promising results. However, the heterogeneous purity of the studied supplements, combined with the variety of physical tests employed call for more studies to confirm these findings, ideally with standardised supplements. Our aim was to test a supplement highly concentrated in DHA (DHA:EPA ratio equal to approximately 8:1) on a maximal cycling test to elucidate performance improvements mainly due to DHA.

**Methods:**

A double-blind, placebo controlled, randomised balanced, parallel design, in competitive amateur cyclists was employed. They were all male, older than 18 years old, with training routine of 2 to 4 sessions per week lasting at least one hour each. A ramp cycling test to exhaustion with a subsequent 5 min recovery phase was employed before and after treatment to analyse aerobic metabolism and lactate clearance after the bout. After 30 days of supplementation with 975 mg of re-esterified DHA, the thirty-eight cyclist who completed the study were finally included for statistical analysis.

**Results:**

Mean power output at ventilatory threshold 2 (VT2) improved after DHA supplementation both as absolute (△DHA versus △PLA: 6.33–26.54 Watts; CI 95%) and relative (*p*=0.006) values, paralleled with higher oxygen consumption at VT2 both for absolute (DHA 2729.4 ±304.5, 3045.9 ±335.0; PLA 2792.3 ±339.5, 2845.5 ±357.1; *ml*·*min*^−1^ baseline versus post *p*=0.025) and relative values (DHA 36.6 ±5.0, 41.2 ±5.4; PLA 37.2 ±5.7, 38.1 ±5.2; *ml*·*kg*^−1^·*min*^−1^ baseline versus post *p*=0.024). Heart rate recovery rate improved during the recovery phase in the DHA group compared to PLA (*p*=0.005).

**Conclusion:**

DHA is capable of improving mean power output at the ventilatory threshold 2 (anaerobic ventilatory threshold) in amateur competitive cyclists. It is unclear if these findings are the result of the specific DHA supplement blend or another factor.

## Background

In the recent years, polyunsaturated fatty acids (PUFAs) have gained ground in the sport field as performance enhancers, with beneficial attributions like improving recovery from injury (by mediation in inflammation pathways [1, 2], both acutely [3, 4] and chronically [5, 6]), muscle soreness [7–13], and improving endurance capacity (by reducing the cost oxygen cost of exercise, consequently lowering heart rate and blood pressure during exercise [14]) and substrate handling (also called as metabolic switching [15]). These properties may be of interest to competitive users, whose training schedule and/or participation in highly damaging events compromises proper recovery (like ultra-endurance competitions [16]) and need a special focus on health [17]. Among them, the most studied forms in sports are eicosapenaenoic acid (EPA) with 5 double bonds (20:5) and docosahexaenoic acid (DHA) with 6 double bonds (22:6).

Additionally, other cardiorespiratory benefits have been addressed for endurance sports such as decreased submaximal and peak heart rate, as well as oxygen consumption during exercise [18], resting heart rate variability [19], submaximal and resting heart rate, systemic vascular resistance, and diastolic blood pressure [20]. Sport performance improvements in endurance sports by DHA and EPA is mainly attributed to their ability to modulate energetic pathways during physical activity, defined as metabolic switching (cell’s ability to switch between substrate utilization [2, 15, 21–23]), which can improve the ability to spare glucose (which is in lower concentrations [24]) and increase fatty acids (FAs) oxidation enabling muscle cells to yield more energy. This is achieved by an increased insulin sensitivity (as observed in humans [15] and in vitro studies with the same human myotube cells [25, 26]). Based on these studies, n-3 PUFAs appears to be a potent stimulator of metabolism in muscle cells and a potential ergogenic aid [27, 28]. However, not all Omega-3 PUFAs sources contain the same amount of bioactive substances, thus they may have different physiological effects.

Dietary omega-3 PUFAs from food sources have a naturally heterogeneous lipid profile: Vegetables oils with high PUFAs content (especially seeds like flaxseed, hemp and wallnuts [29]) may vary their composition according to its environment [30, 31] (probably to protect from oxidation through increased antioxidant activity) while fish species rich in omega-3 (especially cold fishes such as sardines, salmon, tuna, mullet and mackerel [32] with high fat reserves—where the EPA and DHA is accumulated) also present variability even within the same fish specie due to its origin [33] (e.g. farm salmon (*Salmo salar*L.) presented a significantly higher n-3 to n-6 ratio than the regular salmon flesh). The ergogenic study of these omega-3 sources present additional difficulties: A)Vegetable oils are mainly composed of alpha-linolenic acid (ALA)—with variable conversion rate in humans to EPA, on the first conversion step, which then has to be converted DHA, on a second step, by the enzyme omega-3 desatturase (which adds the additional double bond required to the C15) whose activity in human is not very high [14]: resulting in heterogeneous conversion rates ranging from non-significant changes (as observed in the majority of studies [34]) to at most a 38% conversion in an isolated study [35]; B)Nutritional value of fish is not properly assessed by easily observable parameters like colour vivacity (which is due to their majoritary red pigments (carotenoids); not paralleled with its lipid content) and in fact depends highly on their marine algae and micro-algae intake—in fact, the initial DHA and EPA producers in the aquatic food chain [36]—which also present fluctuation in w-3 profiles within their species [37, 38].

The difference between dietary EPA and DHA to that of food supplements used in clinical trials, is that they are made from the concentrated oil, which provides a more controlled framework for clinical experimentation. Unfortunately, concentration in each of them is usually not standardised, therefore not providing further elucidation of the particular physiological contribution by each of them. DHA may exert different qualities compared to EPA in cell function of insulin secreting cells, myocardial and endothelial cells [39], and showed to be more efficient in improving cardiovascular markers like decreased blood pressure, heart rate, platelet aggregation and the ratio between high density lipoproteins (HDL) and low density lipoproteins (LDL) [40]. In the sport nutrition field, recent research showed mixed results in physical performance (see reviews [14, 24, 41]) whose divergent dosing strategy (ranging from low to high doses), diversity of physical protocols employed and heterogeneity in the supplement used make comparisons between results implausible, in addition to an unknown contribution specifically by either EPA and/or DHA to performance outcomes.

The main purpose of this study was to assess exercise performance modifications by DHA supplementation in trained cyclists, primarily as an improvement in aerobic performance markers. We hypothesised that 30 days of supplementation with DHA will improve performance during an incremental cycling test at the sub-maximal intensity. We defined the main variable as power output at ventilatory threshold 2.

## Material and methods

### 2.1 Trial design

A double-blind, placebo controlled, randomised, balanced parallel design, with two different study arms: treatment [docosahexaenoic-acid (DHA)] and placebo (PLA) was employed. Simple randomisation was performed using software (Epidat 4.2, 2016) which generated random codes assigned to participants. An initial incremental exercise test to exhaustion (IETE) was carried out, for baseline assessment of the physical condition of each participant. Afterwards, supplementation was conducted for 1 month (30 days) subsequently performing a second exercise bout with same characteristics, to analyse the effect of the treatment to physical performance.

### 2.2 Subjects

Thirty-eight male amateur cyclists competing at regional level (Region of Murcia, Spain) volunteered to participate in the study. Inclusion criteria were: 1)Male older than 18 years old; 2)Amateur cyclist, with a training routine of 2 to 4 sessions per week, with at least one hour per session; 3)Familiarised with incremental cycling test (IETE). Exclusion criteria were: 1)Allergy to fish or any of its by-products; 2)Serious clinical pathology or antecedents; 3)Undertaking pharmacological treatment 4)Supplementation with omega-3 FA’s as food supplement or as functional food in the last month; 5)Manifestation of any contraindicative symptom during the initial physical assessment according to ACSM/AHA [42]. Participants were informed (verbally and written) of the purpose of the study, the characteristics of product used for the supplementation, its effects, the procedures of the study as well as any possible risk and side effect involved from the supplement. Subjects were informed of their right to quit the study at any time, without the need to provide any reason. Participants gave written consent before the study was started. The study protocol and informed consent were approved by the Ethics Committee of the San Antonio Catholic University of Murcia (UCAM) and were in agreement with the Declaration of Helsinki [43]. To check baseline physical conditioning of both groups, a student-t test was performed for the maximal oxygen consumption obtained after the first incremental test (prior to supplementation).

### 2.3 Supplementation protocol

#### 2.3.1 Product and dosing strategy

Volunteers ingested 3 soft-gels of DHA (BRUDY PLUS, BRUDYTECHNOLOGY, Barcelona, Spain) or a PLA (placebo of sunflower oil) provided by the same manufacturer, being both products identical in appearance. Composition per soft-gel of DHA was: DHA 325 mg, EPA 40 mg, total Omega-3 content 405 mg and a total fatty acid content of 500 mg. Total daily dose was 975 mg of DHA, 120 mg of EPA, 1.22 g of omega-3 PUFAs and 1.50 g of total fat, consumed in a single dose in the morning before breakfast, for 1 month (30 days), which comprised the content of the package (90 softgels). Participants were asked to return the empty packs to ensure compliance. For the follow-up, participants were reminded verbally to consume the supplements.

#### 2.3.2 Initial dietary assessment

A nutritionist performed an initial prospective 24-h dietary recall [44, 45] to assess participants’ diet one week before the initial assessment. Afterwards, a 7-day food record with qualitative and quantitative data [46] along with a printed-guide for proper filling was given to them to calculate daily average intake through the first week of the treatment, and calculated using software (Cronometer Software Inc., https://cronometer.com). This process was repeated on the second visit. Food analysis included the following data: Total energy (expressed as kcal/day) and macro-nutrients (carbohydrates, protein and total fat, expressed in grams/day). Total fat intake was further broken down as saturated, monounsaturated and polyunsaturated fatty acids, as well as *n-*6 and *n-*3 PUFAs were also calculated. In addition, participants were told not to change their usual diet during the study. Subjects were blinded about the results of the dietary analysis to prevent any influence to their dietary habits.

### 2.4 Exercise test

Every cyclist performed two identical maximal efforts with same equipment (calibrated before each test) at the beginning, and after treatment (spaced one month: 30 days). The first one was performed to establish baseline performance values and the second to obtain performance data about the intervention effect on both groups. After each maximal effort, a recovery phase was conducted to obtain data for lactate clearance.

#### 2.4.1 Incremental ramp test to exhaustion

A maximal cycling test was performed to measure performance outcomes. This was composed of an incremental exercise test to exhaustion (IETE) uninterrupted by a recovery phase.

To ensure repeatability of the two trials, every participant used their own bicycle (placed on the rear cassette) and the following measures were employed (as described elsewhere [47]): 1)Front-rear slope-ratio was corrected to zero (using a front wheel riser) during the trial; 2)Bike configuration (gear set, saddle and handlebars) should be kept during the study; 3)Bike fitting (seat-post height and angle, handlebar reach, height and grip position) should be the same and; 4)Preferred pedalling system (use of cycling shoes and type of clip/cleat) should be consistent.

The incremental ramp test consisted of a 3 min warm-up at a self-paced intensity and cadence, followed by an IETE (initial load: 50 Watts (W), with a 5 W increment every 12 s ^−1^) on an electronically braked cycle ergometer (Cyclus2, RBM elektronik-automation GmbH, Leipzig, Germany) at a self-selected cadence between 60-100 revolutions per minute (RPM) which had to be maintained during the whole test. This type of protocol was previously stated as reliable to detect ventilatory threshold 2 (VT2), heart rate (HR) and maximum oxygen consumption (VO2*max*) in healthy individuals [48]. Exhaustion was deemed to occur when the subject decided to stop, when pedal cadence dropped 20 RPM below the minimum cadence established (i.e. 40 RPM), or when power output could not be maintained. During the test, volunteers were verbally encouraged by the staff to exert maximal effort.

Heart rate was monitored beat-to-beat using an electrocardiograph, and oxygen consumption (VO2) was collected continuously during this test using an automated breath-by-breath system (Jaeger Oxyconm ProTM, CareFusion, Hchberg, Germany). All measures were analysed using software (LABManager 5.3.0.4, VIASYS Healthcare GmbH, Hchberg, Germany) and were stored in a personal computer for later recall. Maximal criteria were interpreted according to [49], defined as a plateau of VO2 and heart rate (HR) above 95% of the theoretical maximum HR.

#### 2.4.2 Recovery phase and lactate clearance

After the IETE bout, a subsequent recovery phase was performed for 4 min and 30 s. Constant load was of 50 W and micro-capillary blood (125 *μ*L collected by lancing the left ring-finger pad) were obtained for lactate analysis just at the beginning, 1 min 30 s, 3 min, and 4 min 30 s thereafter. Samples were immediately analysed by a blood gas analyser (ABL90FLEX, Radiometer Medical APS, Copenhagen, Denmark). Oxygen consumption and heart rate was parallelly collected to analyse cardiorespiratory evolution during the recovery phase.

### 2.5 Variables and measurements

During the IETE, the following variables were measured: mean power output (MPO), oxygen consumption (VO2), maximum oxygen consumption (VO2*max*) and heart rate (HR). Ventilatory aerobic and anaerobic threshold were plotted in a graph by using previously mentioned software and interpreted according to the three-phase model [50] by ventilatory equivalents (VE) [51]. VT2 was set as the intersection point between the carbon dioxide ventilatory equivalent (VE/VCO2) and the oxygen ventilatory equivalent (VE/VO2) against time—defined as the point in which pulmonary ventilation during exercise (VE) starts to increase at a faster rate than oxygen uptake (VO2). Time values to reach VT2 were provided by the same software when a vertical line was placed on this intersection point.

### 2.6 Statistical analysis

Sample size was calculated according to the main variable: power output at ventilatory threshold 2, according to its previously described standard deviation of 36.9 W [52] and establishing a precision of 25 W, an alpha risk of 5% and a power of 80%, resulting in a value of n=25 subjects per group. Quantitative variables are described as the mean with standard deviation. This description was made for the total sample and was stratified by the randomised treatment arm. Results are presented in tabular form, including the relative and absolute frequencies for the treatment groups. Data were checked prior to analysis; in all cases, the Kolmogorov-Smirnov test was applied to test for normal distribution, and Levenes test was used to test for homoscedasticity. The evolution of these quantitative variables were analysed by parametric tests: a two-way repeated measures ANOVA test with two Intra-subject factor (product) and one Inter-subject factor (time) for the variables obtained in the IETE. For the *post hoc* group comparison, the Bonferroni test was employed. Statistical analysis was performed using SPSS (version 21.0) and p values are reported for every group and group · time interaction; p < 0.05 is considered statistically significant.

## Results

### 3.1 Participant flow diagram, baseline characteristics and nutritional analysis

**Fig. 1:**
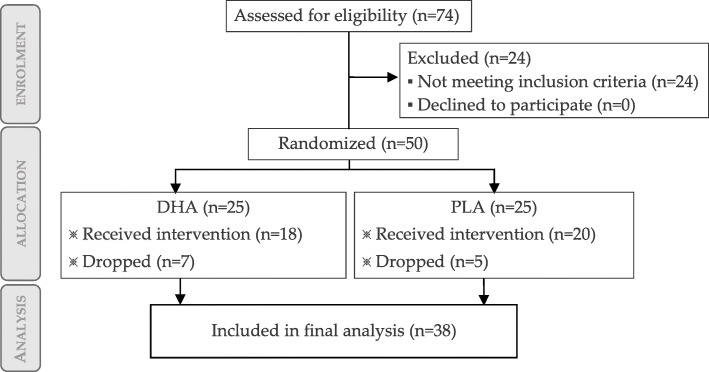
Flow chart of participants

The participant flow diagram is depicted in Fig. 1.

Baseline characteristics of participants were similar for both treatment groups. Placebo (PLA) group characteristics, expressed as mean with standard deviation, were 36.0±9.6 years, weight 71.1±3.4 kg, body mass index 23.42±1.31, absolute VO2 max 3643±362 mL/min, relative VO2 max 48.5±6.8 mL/min/kg. DHA group characteristics were 35.5±7.3 years, weight 72.4±4.4 kg, body mass index 23.83±1.43, absolute VO2 max 3724±341 mL/min, relative VO2 max 50.3±5.2 mL/min/kg. Average of both groups together was 35.8±8.5 years, absolute VO2 max 3682±349 mL/min, relative VO2 max 49.3±6.1 mL/min/kg. Inter-group comparison for relative maximal oxygen consumption for both groups revealed no significant differences at baseline (*p*=0.268). Thus, we assumed similar physical conditioning before intervention. All subjects finished successfully the supplementation protocol. The 7-day food record showed that macro-nutrient values were similar between groups, for every nutritional variable (energy, protein, carbohydrates and total fat). Total fat breakdown also showed homogeneity (saturated, monounsaturated and polyunsaturated fat, further specified as omega-6, omega-3 and DHA+EPA). See Table 7 for a detailed analysis. Therefore we assumed that diet habits were preserved during the study.

**Table 7:** Average daily macro-nutrient intake during intervention for both groups

**Group**	**Energy (Kcal)**	**Carbohydrates (g)**	**Protein (g)**	**Fat (g), of which**	**saturated (g)**	**MUFA (g)**	**PUFA (g)**	**omega-6 (g)**	**omega-3 (g)**	**DHA+EPA (g)**
DHA	2147±400	258±65	94.0±23.8	82.0±29.5	23.9±7.7	37.9±17.1	10.9±5.3	3.2±2.4	0.59±0.53	0.206±0.212
PLA	2218±390	259±52	93.6±21.8	89.4±22.7	26.6±8.2	41.9±12.6	11.8±6.7	3.3±1.3	0.55±0.85	0.194±0.213

Values are presented as mean ± standard deviation. MUFA: monounsaturated fatty acids; PUFA:polyunsaturated fatty acids; DHA: docosahexaenoic acid; EPA: eicosapentaenoic acid

### 3.2 Performance data and cardiorespiratory variable analysis

**Fig. 2:**
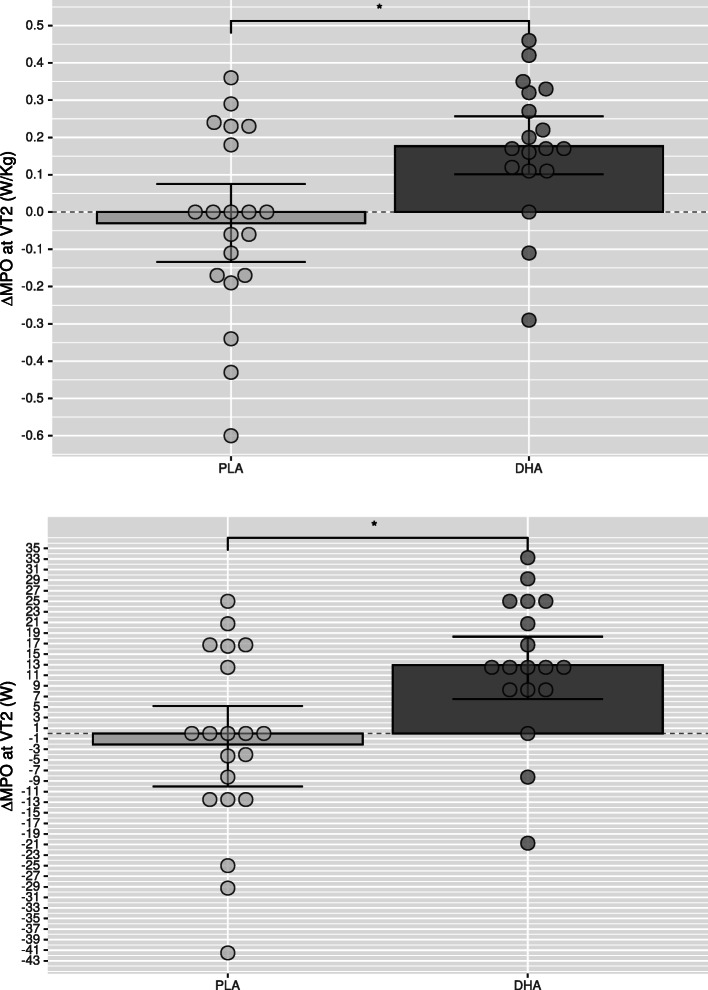
Change of mean power output (MPO) in Watts (W) at ventilatory threshold 2 (VT2) of the DHA (docosahexaenoic acid) group compared to PLA (placebo) group as relative power (upper graphic) and absolute power (lower graphic). Error bars express 95% of confidence interval. * p<0.05

**Fig. 3:**
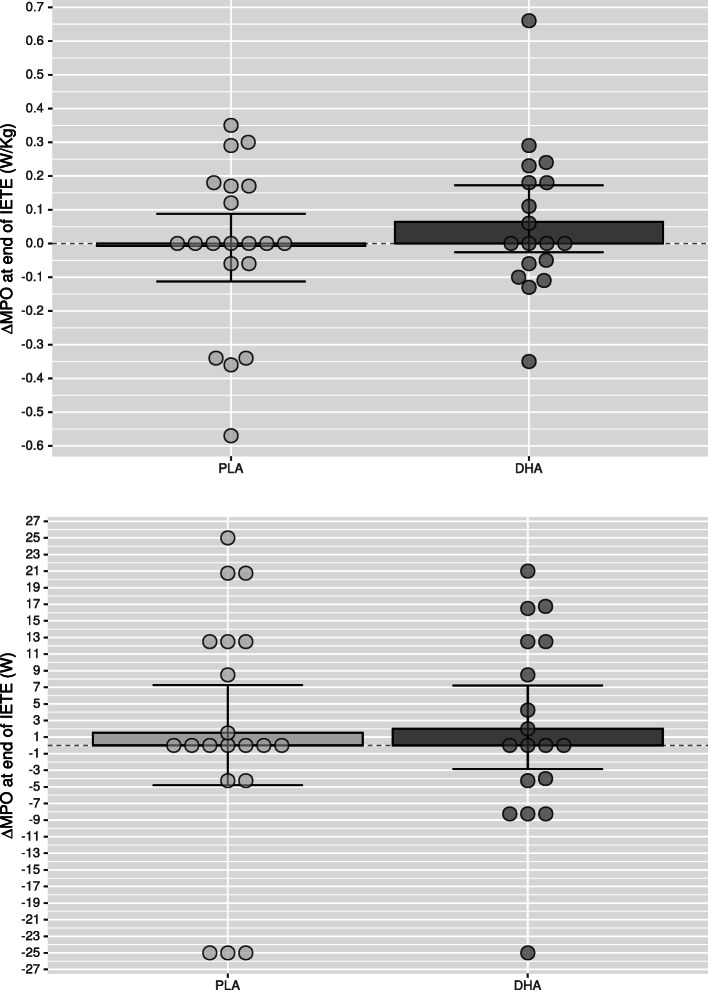
Change of mean power output (MPO) in Watts (W) at the end of the incremental test to exhaustion (IETE) of the DHA (docosahexaenoic acid) group compared to PLA (placebo) group as relative power (upper graphic) and absolute power (lower graphic). Error bars express 95% of confidence interval. * p<0.05

Compared to Placebo, mean power output at VT2 in DHA group was significantly higher for absolute (*p*=0.005,△DHA versus △PLA: 6.33–26.54 W, CI 95%) and relative (*p*=0.006) values after supplementation (Table 4 and Fig. 2) as well as oxygen consumption (VO2) at VT2, both for absolute (*p*=0.025) and relative (*p*=0.024) values (Table 2), which was paralleled with a higher absolute (*p*=0.047) and relative (*p*=0.050) oxygen consumption at minute six (Table 1) and a higher cumulative oxygen consumption during the recovery phase (Table 6). The ventilatory equivalent for oxygen showed intra-group differences in the DHA group (*p*=0.013) without intergroup differences (*p*=0.084; Table 2). No significant differences were found between groups for maximal performance values (Table 5 and Fig. 3), or blood lactate clearance in the recovery phase (Table 6). The DHA group showed a lower HR at minute six (*p*=0.002; Table 1) but not at VT2 (*p*=0.756; Table 2) or the end of the incremental test (*p*=0.172; Table 3), although intra-group analysis in the DHA group reached significance (*p*=0.008; Table 3, Mean change 0.721–4.529; 95% CI). In the recovery phase (Table 6) intra-group HR recovery improved in both groups (PLA: *p*=0.024; DHA: *p*=0.001), showing a greater improvement in the DHA group when compared to placebo (*p*=0.005).

**Table 2:** Cardiorespiratory data at ventilatory threshold 2

**Group**	**Trial**	**Absolute VO**_**2**_$^{\left (ml \cdot min^{-1}\right)}$	**Intra-group*p*-value**	**Relative VO**_**2**_$^{\left (ml \cdot kg^{-1} \cdot min^{-1}\right)}$	**Intra-group*p*-value**	**VEO**_**2**_** (VE/VEO**_**2**_**)**	**Intra-group*p*-value**	**Heart rate (bpm)**	**Intra-group*p*-value**
DHA	*Baseline*	2729±305	} p = 0.001*	36.6±5.0	} p = 0.001*	26.83±2.30	} p = 0.013*	160±11	} p = 0.683
	*Post*	3046±335		41.2±5.4		28.50±2.40		160±13	
PLA	*Baseline*	2792±340	} p = 0.503	37.2±5.7	} p = 0.418	27.85±3.43	} p = 0.870	162±12	} p = 0.242
	*Post*	2846±357		38.1±5.2		27.95±2.37		163±12	
Inter-group · Time ^−1^		p = 0.025*		p = 0.024*		p = 0.084		p = 0.756	

VE/VEO_2_: Ventilatory equivalent for oxygen. Values are presented as mean ± standard deviation (SD). * p <0.05

**Table 5:** Performance data at the end of the IETE

**Group**	**Trial**	**Mean power output (Watts)**	**Intra-group*p*-value**	**Relative power (W/Kg)**	**Intra-group*p*-value**	**Time (minutes’ seconds”)**	**Intra-group*p*-value**
DHA	*Baseline*	373.46±27.89	} p = 0.662	5.14±0.54	} p = 0.251	12^′^56^′′^±1^′^07^′′^	} p = 0.542
	*Post*	375.46±25.72		5.20±0.48		13^′^11^′′^±1^′^17^′′^	
PLA	*Baseline*	346.69±34.48	} p = 0.542	4.92±0.57	} p = 0.896	11^′^52^′′^±1^′^23^′′^	} p = 0.622
	*Post*	348.21±38.99		4.91±0.62		11^′^56^′′^±1^′^34^′′^	
Inter-group · Time ^−1^		p = 0.916		p = 0.355		p = 0.916	

Values are presented as mean ± standard deviation (SD). * p <0.05

**Table 6:** Course of cardiorespiratory variables during the recovery phase

**Variable**	**Group**	**Trial**	**Time (minutes’ seconds”)**				**Cumulative AUC**	**Intra-group*p*-value**	**Inter-group · Time**^**−1**^
			**0**^***′***^**00**^***′′***^	**1**^***′***^**30**^***′′***^	**3**^***′***^**00**^***′′***^	**4**^***′***^**30**^***′′***^			
Lactate ^(*mmol*/*L*)^	DHA	*Baseline*	12.0±3.2	12.4±1.8	11.8±1.7	11.3±1.4	880.6±100.4	} p = 0.841	} p = 0.694
		*Post*	11.2±1.9	12.1±1.9	12.0±2.0	11.2±2.3	874.5±141.6		
	PLA	*Baseline*	11.4±1.7	12.2±2.0	12.0±1.7	11.5±2.1	908.1±157.2	} p = 0.718	
		*Post*	11.3±2.4	12.4±1.6	12.2±1.8	12.1±1.9	918.4±127.0		
VO_2_^(*ml*/*min*)^	DHA	*Baseline*	3662±425	2122±451	1767±363	1556±285	584,825±91,516	} p = 0.034*	} p = 0.136
		*Post*	3662±549	2248±421	1960±365	1750±374	622,230±97,050		
	PLA	*Baseline*	3629±403	2154±336	1791±287	1654±282	592,765±75,399	} p = 0.920	
		*Post*	3600±432	2142±427	1800±407	1726±399	594,396±100,112		
HR (bpm)	DHA	*Baseline*	184±9	158±12	158±12	141±12	41,010±2,778	} p = 0.001*	} p = 0.005*
		*Post*	182±9	151±12	130±11	122±13	38,955±2,719		
	PLA	*Baseline*	181±10	155±13	137±12	137±12	40,259±3,135	} p = 0.024*	
		*Post*	180±10	152±12	134±11	128±9	39,535±2,682		

Values are presented as mean ± standard deviation (SD). Intra-group and inter-group analysis corresponds to the cumulative area under the curve (AUC) * p <0.05

**Table 1:** Cardiorespiratory data at minute 6

**Group**	**Trial**	**Absolute VO**_**2**_$^{\left (ml \cdot min^{-1}\right)}$	**Intra-group*p*-value**	**Relative VO**_**2**_$^{\left (ml \cdot kg^{-1} \cdot min^{-1}\right)}$	**Intra-group*p*-value**	**Heart rate (bpm)**	**Intra-group*p*-value**
DHA	*Baseline*	2376±262	} p = 0.034*	32.9±3.9	} p = 0.041*	152±10	} p = 0.001*
	*Post*	2246±242		31.1±3.0		149±11	
PLA	*Baseline*	2210±252	} p = 0.506	31.1±3.9	} p = 0.636	153±11	} p = 0.864
	*Post*	2248±365		31.5±4.5		153±11	
Inter-group · Time ^−1^		p = 0.047*		p = 0.050*		p = 0.002*	

Values are presented as mean ± standard deviation (SD). * p <0.05

**Table 3:** Cardiorespiratory data at the end of the IETE

**Group**	**Trial**	**Absolute VO**_**2**_$^{\left (ml \cdot min^{-1}\right)}$	**Intra-group*p*-value**	**Relative VO**_**2**_$^{\left (ml \cdot kg^{-1} \cdot min^{-1}\right)}$	**Intra-group*p*-value**	**Heart rate (bpm)**	**Intra-group*p*-value**
DHA	*Baseline*	3724±341	} p = 0.669	50.3±5.2	} p = 0.541	186±8	} p = 0.008*
	*Post*	3719±507		50.4±6.6		183±9	
PLA	*Baseline*	3643±362	} p = 0.949	48.5±6.8	} p = 0.782	182±9	} p = 0.334
	*Post*	3611±399		48.3±6.3		181±10	
Group · Trial · Time ^−1^		p = 0.801		p = 0.859		p = 0.172	

Values are presented as mean ± standard deviation (SD). * p <0.05

**Table 4:** Performance data at ventilatory threshold 2

**Group**	**Trial**	**Mean power output (Watts)**	**Intra-group*p*-value**	**Relative power (W/Kg)**	**Intra-group*p*-value**	**Time (minutes’ seconds”)**	**Intra-group*p*-value**
DHA	*Baseline*	284.42±31.28	} p = 0.001*	3.95±0.50	} p = 0.001*	9^′^23^′′^±1^′^15^′′^	} p = 0.001*
	*Post*	297.38±29.48		4.12±0.47		9^′^54^′′^±1^′^11^′′^	
PLA	*Baseline*	274.10±34.75	} p = 0.555	3.86±0.51	} p = 0.562	8^′^58^′′^±1^′^23^′′^	} p = 0.555
	*Post*	272.03±34.64		3.83±0.51		8^′^53^′′^±1^′^23^′′^	
Inter-group · Time ^−1^		p = 0.005*		p = 0.006*		p = 0.005*	

Values are presented as mean ± standard deviation (SD). * p <0.05

## Discussion

The primary finding is that supplementation with DHA improved aerobic capacity through an increased mean power output at the ventilatory threshold 2, which reflects a higher power output at the same metabolic cost. In parallel, oxygen consumption was increased both as absolute and relative values.

A higher mean power output at the ventilatory threshold 2 is translated into a better aerobic capacity, which is a usual sign of adaptation to a given sport. The population sample was already trained to minimise a high contribution of the training plan during the experiment to this outcome, so the results suggests this effect was triggered mainly by DHA. Further studies with highly trained cyclists should be conducted to confirm this results. Implications of such improvement can be of interest for competitive cyclists in high intensity, short to mid duration races, and in high intensity bouts like climbing segments.

To the best of our knowledge, this study was the first to find positive results in improving VT2 values in a re-esterified fish oil supplement with a high DHA:EPA ratio (8:1). Other studies in cycling which employed regular fish oils (with heterogeneous concentrations of DHA and EPA), found a reduction in oxygen cost during a capacity cycling trial (time trial) [53] and sub-maximal test (55% of VO2 max) [18], while others did not found improvement in cycling trial time (time to complete 70% of maximum work: 70% of WMax) [54], steady sub-maximal test [55] or an incremental test to exhaustion (with steps of 30 W every minute) [56].

In a such a short bout, an improved neurotransmitancy could be a mechanism for the observed improved physical performance. DHA is the essential constituent of neuronal membrane phospholipids, fundamental for neural pathways. Alterations in the membrane composition and fluidity may accelerate conductance of potential actions, increasing motor unit firing rate into the sarcolema [57]. Other studies found that neuromuscular recruitment and strength was improved (after 21 days of n-3 PUFAs suplementation in athletes) [58], together with perceptual-motor benefits by improvements in complex reaction time and efficiency (after supplementation of 3.5 g of DHA rich fish for 4 weeks) in female elite soccer players [59], while another found improved strength (one-repetition maximum in lower and upper limbs) [60].

To our best knowledge, the supplement used in this study (Tridocosahexaenoine-AOX ^Ⓡ^) possess a different composition compared to other fish oils previously studied in sports nutrition. It is obtained by enzymatic synthesis from tuna fish oil [61] and composed of almost only DHA in triglyceride form (representing 70% of total fatty acids and 90% of total omega-3 PUFAs) after re-esterification, with a high proportion in the second glycerol position (sn-2). Usually natural fish oils contain low amounts of DHA due to the presence of only one DHA molecule in the tryglyceride (in the sn-2 position) but in re-esterified triacylglycierols (rTAG) formulas, a random re-esterification can place another DHA mollecule in the sn-1, sn-3 or both positions, by default occupied by a mid-chain or short-chain FA (hence the high proportion of DHA in the final formula)—and also resulting in diacylglycerides, monoacylglycerides and free FAs [62]. This esteric configuration can be more favourable, and likely have a decisive influence on bioavailability [63]. Triglycerides coming from the diet of fish oils are hydrolised by pancreatic lipases before absorption which acts on the sn-1 or sn-3, to form a monoacylglycerol with the remaining FA in the sn-2 position (2-MAG) and free FA: all absorbed in the form of micelles in the gut lumen with additional steps required until it reaches the cell. Ultimately, incorporation of DHA to cells where it exert a physiological action, better reflects overall bioavailibility of the product. Some studies showed that rTAG are more bioavailable with increased incorporation to erytrocites [64] and other tissues like brain when the DHA was on sn-2 [65]. DHA supplements in the market can come as ethyl esters, monoacylglycerols or regular triglycerides: point which is usually disregarded in DHA clinical trials assessing physical outcomes, and which may have contributed to the disparity of results of previous studies. Future research should consider bioavailability implications of the product employed, and encourage proper assessment of its bioavailability [66] to elucidate more at this respect. 

There may be a contribution by one or more of the mechanisms discussed above by Tridocosahexaenoine-AOX ^Ⓡ^ to our results, as observed by previous work with same DHA triglyceride. These showed increased endogenous antioxidant activity (by induction of intra-cytoplasmic gluthatione synthesis [67]), neuroprotection (independent on incorporation of other PUFA to the brain) [68], mediation in inflammation processes—in neurones [61] and subcutaneous adipose tissue (by down-regulation of inflammatory gene expression), and in systemic inflammation (restricted to high sensitivity C reactive protein and a decrease in arachidonic acid [69])—and by activation of the cellular antioxidant network (probably as a result of an adaptive response of the cell) [70] in animal models. However, as a limitation of our study, we did not measure neurological influence of the product, or a relation of this parameters to our performance outcomes.

As a secondary finding, we did not find maximal performance variables like absolute or relative maximum VO2*max* or mean power output at the end of the incremental test, which is in line with other studies employing submaximal test with fish oil [55] or incremental or submaximal test with omega-3 PUFAs [71, 72].

As another secondary finding, we did not find significant changes in peak heart rate or HR in the ventilatory threshold 2, although intra-group comparisons showed a significant improvement in the DHA group at the end of the incremental test after supplementation. However, a further analysis of HR in the recovery phase, showed that intra-group HR recovery improved in both groups, showing a greater improvement in the DHA group when compared to placebo. In lack of any supplementation, peak HR values are not usually changed in trained population (as shown by a longitudinal study in elite cyclist through the competitive season) in spite of performance improvements as an increased power output at lactate and ventilatory thresholds [73]. Myocardial cells incorporate higher levels of omega-3 PUFAs than red blood cells [74, 75], which may contribute to a more efficient heart functioning during physical activity (thus lowering heart rate) by an improved red blood cell deformability [76, 77] and a reduced myocardial oxygen consumption [78]. Due to the limitations of our study, we did not measured incorporation of DHA and EPA to erythrocyte membranes or heart tissue, which is related to incorporation to other tissues, which showed that at least 30-60 days are required for the heart cells to uptake omega-3 [79]. Experimental results showed heterogeneous outcomes when observing HR in maximal and sub-maximal efforts. In physically active population, peak HR during a maximal effort showed both positive results [18] or no significant results [19, 20], which also was the case for HR during sustained sub-maximal efforts, also with positive findings [18–20, 80] or no significant results [55]. This is in line with clinical trials in patients with some kind of heart disease [81, 82] which showed no improvement in peak HR.

In this study we found that supplementation with re-esterified DHA in amateur cyclists improved the mean power output at ventilatory threshold 2, which is synonymous of an improved aerobic efficacy. Due to the heterogeneity of the outcomes and protocols found in the literature, replication with standardised supplements (purity controlled and properly characterised) and further research tailored to measure performance at this intensity are recommended to confirm these results.

## Conclusion

DHA in amateur cyclist may improve cycling performance by enhancing power output at the anaerobic ventilatory threshold 2. It is unclear if these findings are the result of the specific DHA supplement blend or another factor.

## Data Availability

The dataset analysed in this study will me made available upon reasonable request.
